# A rare 47 XXY/46 XX mosaicism with clinical features of Klinefelter syndrome

**DOI:** 10.1186/s13633-016-0029-3

**Published:** 2016-06-02

**Authors:** Noor Shafina Mohd Nor, Muhammad Yazid Jalaludin

**Affiliations:** Faculty of Medicine, Universiti Teknologi MARA, Sungai Buloh, Selangor Malaysia; Department of Paediatrics, Faculty of Medicine, University of Malaya, 50603 Kuala Lumpur, Malaysia; Paediatric and Child Health Research Group, Faculty of Medicine, University Malaya, Kuala Lumpur, Malaysia

**Keywords:** 47XXY/46XX mosaicism, Klinefelter syndrome, Radioulnar synostosis

## Abstract

**Background:**

47 XXY/46 XX mosaicism with characteristics suggesting Klinefelter syndrome is very rare and at present, only seven cases have been reported in the literature.

**Case presentation:**

We report an Indian boy diagnosed as variant of Klinefelter syndrome with 47 XXY/46 XX mosaicism at age 12 years. He was noted to have right cryptorchidism and chordae at birth, but did not have surgery for these until age 3 years. During surgery, the right gonad was atrophic and removed. Histology revealed atrophic ovarian tissue. Pelvic ultrasound showed no Mullerian structures. There was however no clinical follow up and he was raised as a boy. At 12 years old he was re-evaluated because of parental concern about his ‘female’ body habitus. He was slightly overweight, had eunuchoid body habitus with mild gynaecomastia. The right scrotal sac was empty and a 2mls testis was present in the left scrotum. Penile length was 5.2 cm and width 2.0 cm. There was absent pubic or axillary hair. Pronation and supination of his upper limbs were reduced and x-ray of both elbow joints revealed bilateral radioulnar synostosis. The baseline laboratory data were LH < 0.1 mIU/ml, FSH 1.4 mIU/ml, testosterone 0.6 nmol/L with raised estradiol, 96 pmol/L. HCG stimulation test showed poor Leydig cell response. The karyotype based on 76 cells was 47 XXY[9]/46 XX[67] with SRY positive. Laparoscopic examination revealed no Mullerian structures.

**Conclusion:**

Insisting on an adequate number of cells (at least 50) to be examined during karyotyping is important so as not to miss diagnosing mosaicism.

## Background

Klinefelter syndrome is relatively common and affects 1:500 to 1:1000 male live births [1]. This syndrome is a group of chromosomal disorders in which there is at least one extra X chromosome added to the normal 46XY male karyotype [1]. It is characterized by hypogonadism, gynaecomastia, azoospermia or oligospermia, and increased levels of gonadotropins [2]. Variants of Klinefelter syndrome are rare and occur when more than one extra sex chromosome is present in each cell, eg 48,XXXY or 49,XXXXY. In addition to affecting male sexual development, variants of Klinefelter syndrome are also associated with an increased risk for other congenital malformations, additional medical problems and complex psychological involvement [3].

Mosaicism in Klinefelter syndrome has also been documented with 47,XXY/46,XY karyotype affecting about 10 % of cases [4]. They tend to have milder signs, depending on the number of cells with an additional X chromosome. When a mosaic for a 46,XY cell line is present, it is usually associated with a variety of fertility-associated problems, ranging from azoospermia to different grades of testicular insufficiency [5]. On the other hand, 47 XXY/46 XX mosaicism with characteristics suggesting Klinefelter syndrome is very rare and to date, only seven cases have been reported in the literature [5–11].

## Case presentation

We report a 15-year old Indian boy who was brought to the clinic (at age 12 years) by his parents who were concerned about his body shape which had female fat distribution. He was born in a private hospital and noted to have right cryptorchidism and chordae at birth, but did not have surgery for these until age 3 years. During surgery, the right gonad was found to be atrophic and hence removed. Histology revealed atrophic ovarian tissue. Pelvic ultrasound at the time showed no Mullerian structures. Unfortunately, he was lost to follow-up and was continued to be raised as a boy. He had three other brothers who were healthy. His development was not delayed, he had no learning difficulties and had an average academic performance.

Physical examination at our hospital when he was re-evaluated at 12 years old revealed his weight was 44.1 kg (+0.42 SDS), height 148.4 cm (−0.32 SDS, MPH 175 cm, −0.21 SDS), arm span of 147.8 cm, body mass index 20.2 kg/m2 (+1.03 SDS) with broad hips. There was mild gynaecomastia (similar to breast Tanner stage 3) but no galactorrhoea. His right scrotum was empty and there was only a palpable 2mls testis in the left scrotum. Penile size was 5.2 cm × 2.0 cm with no pubic or axillary hair. Examination of both his upper limbs revealed reduced range of movement on pronation and supination which led him to having difficulty in opening bottle-caps and door-knobs. The rest of his physical examination was normal.

**Fig. 1:**
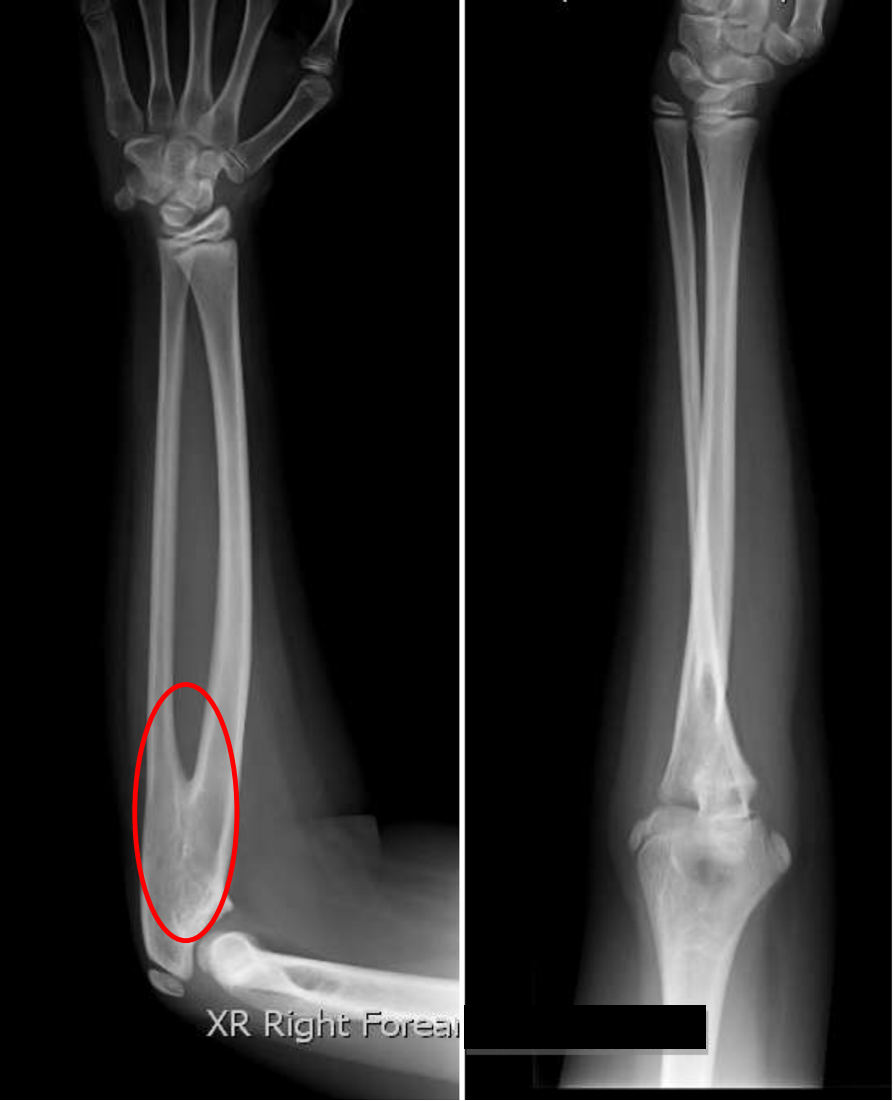
X-ray of right forearm

X-ray of the right forearm including the elbow joint showed a proximal radio-ulnar fusion/synostosis (Fig. 1). The bone age was 12 years, similar to his chronological age.

Baseline laboratory data were LH < 0.1 mIU/ml, FSH 1.4 mIU/ml and testosterone 0.6 nmol/L with estradiol 96 pmol/L. HCG stimulation test showed a minimal rise of testosterone from 0.6 nmol/L on day 0 to 3.0 nmol/L on day 4. Chromosome study based on 76 cells revealed 47 XXY [9, 12 %]/46 XX [67, 88 %] with positivity of SRY determined by FISH. Laparoscopic examination of the abdomen and pelvis confirmed absence of Mullerian structures with small left testis within the scrotal sac, epididymis and vas deferens. He received 50 mg intramuscular testosterone injection every 4-weeks at the age of 13 years to initiate his pubertal development. At the age of 14.5 years, his repeat baseline laboratory data taken on the day of intramuscular testosterone 100 mg every 4-weeks, showed LH 6.3 mIU/ml, FSH 9.2 mIU/ml, testosterone 4.3 nmol/L, consistent with partial hypergonadotropic hypogonadism.

## Discussion

Apart from being rare, mosaic 47 XXY/46 XX has variable phenotypes and clinical presentations. Following an exhaustive literature review, to date there were only seven reported cases of true 47 XXY/46 XX with clinical features of Klinefelter syndrome [5–11]. To the best of our knowledge, our case is the eighth case in the current literature. 47 XXY/46 XX mosaicism were also reported to present as ovotesticular disorder of sexual development (DSD) [12–20] or normal female phenotype [21].

In contrast to our case, patients with 47 XXY/46 XX mosaicism with clinical features of Klinefelter syndrome mostly present at an older age with age ranging from 14.5 to 62 years old [5, 8, 9, 11]. However, 47XXY/46XX, ovotesticular disorder of sexual development had been documented in a 5-month old infant boy with hypospadias, unilateral cryptorchidism and micropenis. Ovarian tissue, fallopian tube and remnants of the uterus were observed in the left inguinal canal whereas testicular tissue was observed in the right scrotum during laparoscopy [14]. It is fortunate that this patient presented to us around his early pubertal years. Otherwise he may have gone undiagnosed and later may have presented with oligospermia or azospermia.

Examination of Klinefelter Syndrome patients usually reveals small testicular size with sparse or absent growth of facial or pubic hair. Unlike 47XXY Klinefelter, IQ was not affected in some cases [5, 22, 23], as seen in our patient, although unfortunately we do not have a formal IQ assessment. The association of 47XXY/46XX mosaicism with broad spectrum of phenotypes is again demonstrated in one case who had a normal female phenotype whereby the cytogenetic investigation revealed that most of the cells had a karyotype of 46 XX and a minority of 47 XXY [21]. On the contrary, our patient demonstrated predominant male phenotype even with 88 % of 46 XX in the leukocytes. Previous literatures have demonstrated that both phenotypic sex and the gonadal phenotype are highly influenced by the percentage and distribution of Y-bearing cells in the gonads, however not necessarily in the patients’ peripheral blood leukocytes [24, 25]. Unfortunately, we do not have the karyotype of the testicular tissue, nevertheless we suspect that, given the patient’s phenotype, most of the remaining gonad had a predominance of Y bearing cells.

In addition, our patient also had bilateral radioulnar synostosis. A review of literature reporting Klinefelter’s syndrome and its chromosomal variations showed 18 cases of proximal radioulnar synostosis [26]. The finding of proximal radioulnar synostosis in a single generation should alert clinicians to suspect a disorder of sex chromosome abnormality [26] and hence karyotyping should be ordered. Recent literature on 40 affected males with 49XXXXY showed a high prevalence of musculoskeletal disorder where 75 % of them had radioulnar synostosis [27]. In addition, James et al. [28] found a mosaic karyotype 46,XY/47,XYY/48,XYYY in a 3-year-old boy with bilateral radioulnar synostosis. Our patient with 47XXY/46XX with clinical features of Klinefelter and radioulnar synostosis adds further to the spectrum of mosaicism in variants of Klinefelter syndrome.

Baseline estradiol level in our patient was noted to be high for a boy. Since he was overweight and had no evidence of adrenarche, the source of high estradiol was attributed to the extra fat tissue. It is well recognized that body fat is a significant extragonadal source of oestrogen [29]. The human chorionic gonadotropin (hCG) test evaluated Leydig cell function and this patient showed an increase of five-fold. However the testosterone level at day four of the test was only 3.0 nmol/L which was inadequate for his age. Patients with Klinefelter syndrome usually have primary gonadal failure with very high gonadotrophins but this was not demonstrated in our patient because he was still in the pre-pubertal age. We suspect that his FSH will increase with age.

## Conclusions

In conclusion, insisting on an adequate number of cells (at least 50) to be examined during karyotyping is important so as not to miss diagnosing mosaicism.

## Ethical approval

We have received the ethical approval by the University Malaya Medical Centre Medical Ethics Committee and the reference number is MECID No 20161-2002.

## Consent

Written informed consent was obtained from the parent for publication of this case report and any accompanying images. A copy of the written consent is available for review by the Editor-in-Chief of this journal.

## Abbreviations

FSH: follicle-stimulating hormone
HCG: human chorionic gonadotropin
LH: luteinizing hormone
SRY: sex-determining region Y
